# In-hospital cardiac arrest: evidence and specificities of perioperative cardiac arrest

**DOI:** 10.1186/s13054-022-04300-w

**Published:** 2023-01-13

**Authors:** Quentin de Roux, Athanasios Chalkias, Theodoros Xanthos, Nicolas Mongardon

**Affiliations:** 1 Service d’Anesthésie-Réanimation Chirurgicale, DMU CARE, DHU A-TVB, Assistance Publique-Hôpitaux de Paris (AP-HP), Hôpitaux Universitaires Henri Mondor, 1 rue Gustave Eiffel, 94000 Créteil, France; 2 U955-IMRB, Equipe 03 “Pharmacologie et Technologies pour les Maladies Cardiovasculaires (PROTECT)”, Inserm Univ Paris Est Créteil (UPEC), Ecole Nationale Vétérinaire d’Alfort (EnVA), 94700 Maisons-Alfort, France; 3 Faculté de Santé, Univ Paris Est Créteil, 94010 Créteil, France; 4 Department of Anesthesiology, Faculty of Medicine, University of Thessaly, 41100 Larissa, Greece; 5 Outcomes Research Consortium, 9500 Euclid Avenue, Cleveland, OH 44195 USA; 6 School of Health Sciences, University of West Attica, 12243 Athens, Greece

To the Editor,

We read with great interest the article by Penketh and Nolan, which provides a comprehensive overview on in-hospital cardiac arrest (IHCA) [1]. We fully concur with the authors that guidelines for treatment of IHCA are lacking, and that several data are extrapolated from out-of-hospital cardiac arrest literature. Despite the extensive evidence from rigorous randomized controlled trials, professional guidelines reflect little tangible progress and a main contributing factor is the stagnation of resuscitation science due to our poor knowledge of the underlying pathophysiological mechanisms [2].

Regarding IHCA, this disheartening situation is more evident during the perioperative period as guidelines for treatment of perioperative cardiac arrest have been for long a major gap of knowledge. However, perioperative cardiac arrest has several specificities that are not detailed in Penketh’s review and that deserve considerations: potential neuroprotective effect of anesthesia drugs, immediate diagnosis in monitored patients, timely treatment by trained anesthesiologists, causes of cardiac arrest mostly related to preoperative complications, complications of anesthesia or complications of surgical procedures and finally, good long-term functional outcome [3].

In order to provide a scientific background for decision-making, as well as a guide for future research on perioperative cardiac arrest, the PERIOPCA Consortium recently published for the first time a consensus on 22 PICO questions specially formulated for the perioperative setting [4]. These recommendations (Table 1) are strengthened by a strict methodology including a modified Delphi consensus-building strategy and can be used in clinical practice.

**Table 1:** Summary of perioperative cardiac arrest (PERIOPCA) recommendations

PICO	Recommendation
ETCO_2_ as a prognosis tool of cardiac arrest	In patients with PERIOPCA, it may be reasonable to maintain an ETCO_2_ ≥ 10 mmHg during advanced life support. However, ETCO_2_ should be evaluated in the context of the patient’s clinical status and individualized targets may be necessary considering the cause of arrest, the degree of hypoxia, the quality of CPR and time to ROSC (COR/LOE: IIb/C-EO)
Monitoring physiological parameters during CPR	In adults with cardiac arrest in the perioperative setting, the use of physiological feedback may be reasonable to increase CPR quality and improve short- and long-term outcome (COR/LOE: IIb/C-EO)
Chest compression or defibrillation strategy for Ventricular Fibrillation (VF) or Pulseless Ventricular Tachycardia (pVT)	In adult patients with PERIOPCA, ventricular fibrillation/pulseless ventricular tachycardia should be defibrillated within 3 min after the onset of the arrest (COR/LOE: I/C-LD). The use of AEDs in patients with ventricular fibrillation/pulseless ventricular tachycardia can be useful for improving survival (COR/LOE: IIa/C-LD). It is not recommended to defibrillate patients with ventricular fibrillation/pulseless ventricular tachycardia lasting more than 3 min without prior chest compressions (COR/LOE: III/C-LD)
Timing of administration of epinephrine	In adult patients with PERIOPCA, epinephrine administration after the 3rd shock can be beneficial (COR/LOE: IIa/C-LD)
Standard-dose epinephrine (SDE) versus low-dose epinephrine (LDE) or high-dose epinephrine (HDE)	In patients with PERIOPCA, it may be reasonable to administer 1 mg epinephrine for improving coronary perfusion pressure (COR/LOE: IIb/C-EO)
No vasopressor versus epinephrine, or vasopressin	In patients with PERIOPCA, it may be reasonable to administer epinephrine every 3–5 min (COR/LOE: IIb/C-EO)
Antiarrhythmic drugs for cardiac arrest	In adult patients with PERIOPCA, it is recommended to administer amiodarone or lidocaine for the treatment of ventricular fibrillation/pulseless ventricular tachycardia (COR/LOE: I/C-LD). Magnesium is not indicated for the treatment of ventricular fibrillation/pulseless ventricular tachycardia in the perioperative setting (COR/LOE: III/C-LD)
Timing of administration of anti-arrhythmic	In adult patients with perioperative ventricular fibrillation/pulseless ventricular tachycardia, it might be reasonable to administer amiodarone or lidocaine after the 3rd shock (COR/LOE: IIb/C-EO)
Ventilation rate during continuous chest compressions	In adult patients with PERIOPCA and a secure airway, a ventilation rate of 10 breaths/min during CPR may be reasonable (COR/LOE: IIb/C-EO)
Cardiac arrest associated with pulmonary embolism	In adult patients with PERIOPCA due to pulmonary embolism or suspected pulmonary embolism, early consideration of thrombolysis and CPR duration of at least 60–90 min with or without the use of a mechanical chest compression device may be reasonable before terminating resuscitation attempts (COR/LOE: IIb/C-LD). The emergency treatment option among fibrinolytic therapy, surgical, or mechanical thrombectomy should be selected based on timing and available expertise, since no clear benefit of one approach over the other has been demonstrated
Cardiac arrest during pregnancy	In pregnant women with PERIOPCA, the effectiveness of any special interventions, compared to standard measures, is uncertain, except probably for manual uterine displacement during chest compressions (COR/LOE: IIb/C-EO). In pregnant women with PERIOPCA due to suspected or proven pulmonary embolism, it may be reasonable to use thrombolysis or other measures to remove clot (e.g., surgical or percutaneous pulmonary embolectomy) (COR/LOE: IIb/C-EO). Extracorporeal membrane oxygenation may be considered as an acceptable salvage therapy for pregnant and postpartum patients with PERIOPCA or those with critical cardiac or pulmonary illness (COR/LOE: IIb/C-EO)
Opioid toxicity	In patients with PERIOPCA due to opioid toxicity, it might be reasonable to administer specific agents in addition to advanced life support (COR/LOE: IIb/C-EO)
Epinephrine, vasopressin, steroids, and their combination during or after CPR	In adult patients with PERIOPCA, it is reasonable to administer corticosteroid or mineralocorticoid or the combination of vasopressin, epinephrine, and steroids during/after CPR to increase ROSC (COR/LOE: IIa/B-R). In these patients, these drugs can be useful for improving survival to discharge with good functional outcome (COR/LOE: IIa/B-R)
Lipid therapy for cardiac arrest	In adult patients with PERIOPCA due to confirmed or suspected LAST, it may be reasonable to use lipid therapy (COR/LOE: IIb/C-LD)
Ultrasound during CPR	In patients with PERIOPCA, it may be reasonable to use point-of-care ultrasound to improve CPR and increase survival rates (COR/LOE: IIb/C-EO)
ECPR versus manual or mechanical CPR	In adult patients with PERIOPCA, it may be reasonable to use ECPR as a rescue therapy when CPR has failed to provide ROSC or non-sustained ROSC (COR/LOE: IIb/C-LD)
Postresuscitation hemodynamic support	In patients with ROSC after PERIOPCA, it may be reasonable to target the hemodynamics goals to optimize tissue perfusion as indicated by an adequate urine output (1 ml/kg/h) and normal or decreasing plasma lactate values, taking into consideration the patient’s normal blood pressure, the cause of the arrest and the severity of any myocardial dysfunction (COR/LOE: IIb/C-EO)
Postresuscitation antiarrhythmic drugs	In the perioperative setting, it may be reasonable to administer antiarrhythmics immediately after ROSC to treat postresuscitation arrhythmias, especially in refractory cases, and prevent recurrences (COR/LOE: IIb/C-EO)
Postresuscitation permissive hypercapnia	In patients with ROSC after PERIOPCA, a lung-protective ventilation strategy (reducing tidal volume, plateau pressure, and driving pressure) and mild hypercapnia (PaCO_2_ of 40–50 mmHg) might be reasonable for improving outcome (COR/LOE: IIb/C-EO)
Postresuscitation target of PaO_2_	In patients with PERIOPCA, it may be reasonable to maintain normoxemia and avoid hyperoxemia (PaO_2_ goal of < 200 mmHg) in order to improve short and long-term outcome (COR/LOE: IIb/C-EO)
Targeted temperature management	In comatose patients with PERIOPCA, it may be reasonable to maintain normothermia in order to improve short and long-term outcome (COR/LOE: IIb/C-EO). Potential neurological benefit should be balanced against the hemorrhagic risk related to hypothermia (< 37 °C) in this surgical setting
Prognostication in comatose patients treated with hypothermic targeted temperature management	In patients with PERIOPCA and ROSC, it may be reasonable to use a multimodal strategy for prognostication, giving emphasis on allowing sufficient time for neurological recovery and to enable sedatives/paralytics to be cleared (COR/LOE: IIb/C-EO)

*PERIOPCA* perioperative cardiac arrest, *CPR* cardio-pulmonary resuscitation, *ECPR* extracorporeal cardio-pulmonary resuscitation, *ROSC* return of spontaneous circulation, *COR* class of recommendation, *EO* expert opinion, *LD* limited data, *LOE* level of evidence

Fortunately, the perioperative setting supports a physiology-guided treatment strategy to titrate the resuscitation efforts to patient’s physiological response. Therefore, translational research should be intensively used as a bridge between different areas of research to improve survival rates. Such an approach is currently investigated in the PERSEUS-PS randomized controlled trial (NCT04428060) [5].

We hope that the PERIOPCA recommendations and the results of the PERSEUS-PS trial will serve as a basis and would be of interest for the International Liaison Committee on Resuscitation or the scientist who wants to build upon the available evidence.

## Data Availability

Not applicable.
